# Pulmonary artery *in situ* thrombosis due to patent ductus arteriosus: a case report

**DOI:** 10.3389/fcvm.2024.1433847

**Published:** 2024-09-10

**Authors:** Yin Wang, Chunyan Rong, Ming Lu, Weihua Zhang

**Affiliations:** Department of Cardiovascular Medicine, The First Hospital of Jilin University, Changchun, China

**Keywords:** pulmonary artery *in situ* thrombosis, congenital heart disease, patent ductus arteriosus (PDA), pulmonary embolism, PAIST

## Abstract

**Background:**

Pulmonary Artery *in situ* Thrombosis (PAIST) refers to a thrombus forming within the pulmonary arterial system, distinct from an embolus originating from elsewhere in the body (e.g., the deep veins of the lower extremities) and traveling to the lungs where it lodges and forms.

**Case presentation:**

We present a case of PAIST caused by the arterial ductus arteriosus. The patient primarily presented with dyspnea, and the chest pain dichotomy Computed Tomography Angiography(CTA) suggested that a nodular low-density filling defect was seen in the lumen of the left pulmonary artery trunk. Initially, pulmonary embolism (PE) was suspected. However, upon reevaluation of the imaging, it became apparent that the patient's pulmonary artery obstruction was closely associated with the ductus arteriosus. After admission, the patient was treated with sodium ampicillin (2.0 g Q12H) for infection, heparin sodium (5,000 IU Q12H) for anticoagulation, and metoprolol succinate extended-release tablets (23.75 mg QD) to correct cardiac remodeling, among other treatments. Subsequently, the patient underwent a cardiac surgery involving the ligation of the arterial duct, resection of pulmonary artery lesions, and open-heart surgery with extracorporeal circulation support. Postoperative examination of the pulmonary artery mass indicated coagulation tissue. The final diagnosis was “PAIST”.

**Conclusion:**

Both PAIST and PE manifest as low-density filling defects in the pulmonary arteries. However, due to the relative unfamiliarity with PAIST, such findings are often initially attributed to PE.

## 1 Background

PAIST typically arises amidst pathophysiologic lung changes, featuring thrombosis within the pulmonary arterial system. This condition often presents with thrombus propagation from the peripheral to the central pulmonary arteries, inducing hemodynamic alterations within the lungs. Consequently, patients may experience symptoms like dyspnea and chest pain, albeit right heart insufficiency is a rare occurrence (1, 2). Various factors, including structural lung changes, infections, trauma, and sickle cell disease, can precipitate focal inflammation and dysfunction of the pulmonary vascular endothelium, thereby fostering the development of PAIST. The precise mechanism underlying its formation remains elusive and may entail pulmonary artery endothelial cell damage, heightened blood viscosity, augmented blood flow, and inflammation (1, 2). A study on a clinical translational model of blunt chest trauma first demonstrated the occurrence of *de novo* pulmonary thrombosis, further supporting the formation of *in situ* pulmonary artery thrombi following severe trauma (3). Literature reports suggest that PAIST seems more likely to occur in patients with acute chest syndrome (ACS) who have elevated platelet counts and lower hemolysis rates (4). PAIST is relatively rare, and the exact incidence is unknown, However, there is growing evidence in the literature suggesting that the incidence of PAIST may have been underestimated, particularly in recent years, as seen in case review studies of COVID-19 patients with concurrent PE, Literature reports indicate that pulmonary artery thrombosis in COVID-19 is an immune-mediated inflammatory thrombosis, with an incidence of pulmonary artery thrombosis/embolism of 36% among COVID-19 patients (5–8). Knudson et al. recently reviewed injury data from 888,652 trauma patients in the National Trauma Data Bank (NTDB) and reported a significant increase in the incidence of PE, while the incidence of deep vein thrombosis (DVT) did not increase (9). Currently, there is no diagnostic gold standard for PAIST, and the absence of specific imaging or pathological manifestations to determine the etiology of thrombosis makes it primarily a diagnosis of exclusion. Imaging tests, however, can be relied upon to aid in the diagnosis (1), such as Computed tomography pulmonary angiography right (CTPA) or Magnetic resonance imaging (MRI).PAIST has a better prognosis and is treated similarly to PE with therapeutic measures such as anticoagulation, thrombolysis, and surgery (1, 2). A retrospective study involving 23 patients with PAT indicated that thrombolysis of *in situ* pulmonary artery thrombi occurs more slowly compared to pulmonary embolism (10). In this paper, we report a case of PAIST suspected to be PE and review the relevant literature.

## 2 Case presentation

The patient, a 59-year-old male, was admitted to the hospital due to “sudden dyspnea persisting for more than half a day”. He experienced a sudden onset of dyspnea without accompanying chest pain, cough, fever, or other discomforts. He had (6–8) previously been in good health. Physical examination on admission revealed the body temperature was 36.5℃, the pulse rate was 99 beats per minute, the breathing rate was 20 beats per minute, blood pressure was 151/100 mmHg (1 mmHg = 0.133 kPa), his spirit was clear and the speech is clear, there was no cyanosis of the lips or mouth, and there was no filling of the jugular veins. Breath sounds were coarse in both lungs, and no dry or wet rales were heard. The apical beat was located between the 5th intercostal space and 0.5 cm outside the left midclavicular line, the cardiac border was enlarged, and a continuous mechanical murmur could be heard in the second intercostal space at the left edge of the sternum, but no murmur was heard in the rest of the heart.

**Figure 1:**
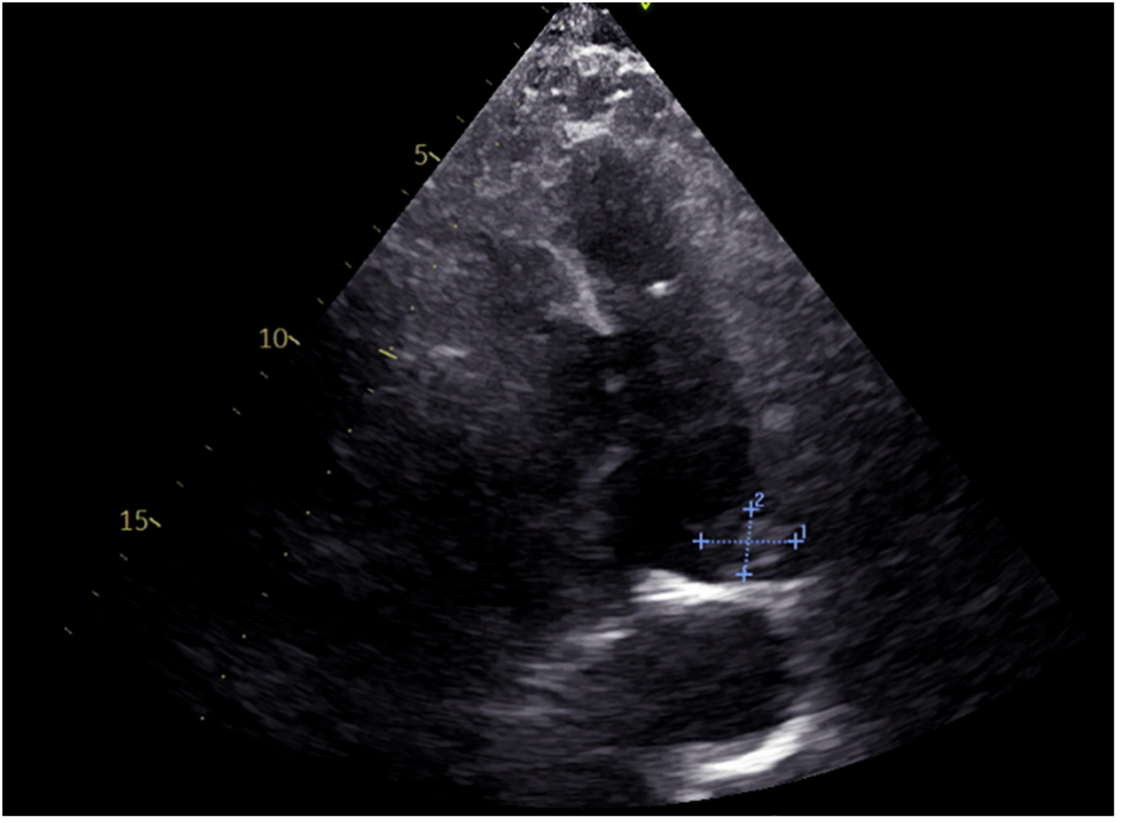
ECG: abnormal echoes of the left pulmonary artery, to be excluded superfluous (21 × 14 mm)?

**Figure 2:**
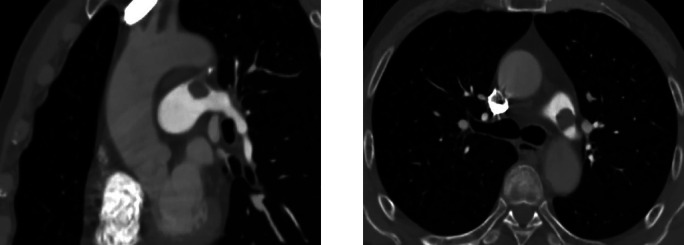
CTA of chest pain diathesis: nodular low-density filling defect in the left pulmonary artery trunk in relation to the ductus arteriosus.

**Figure 3:**
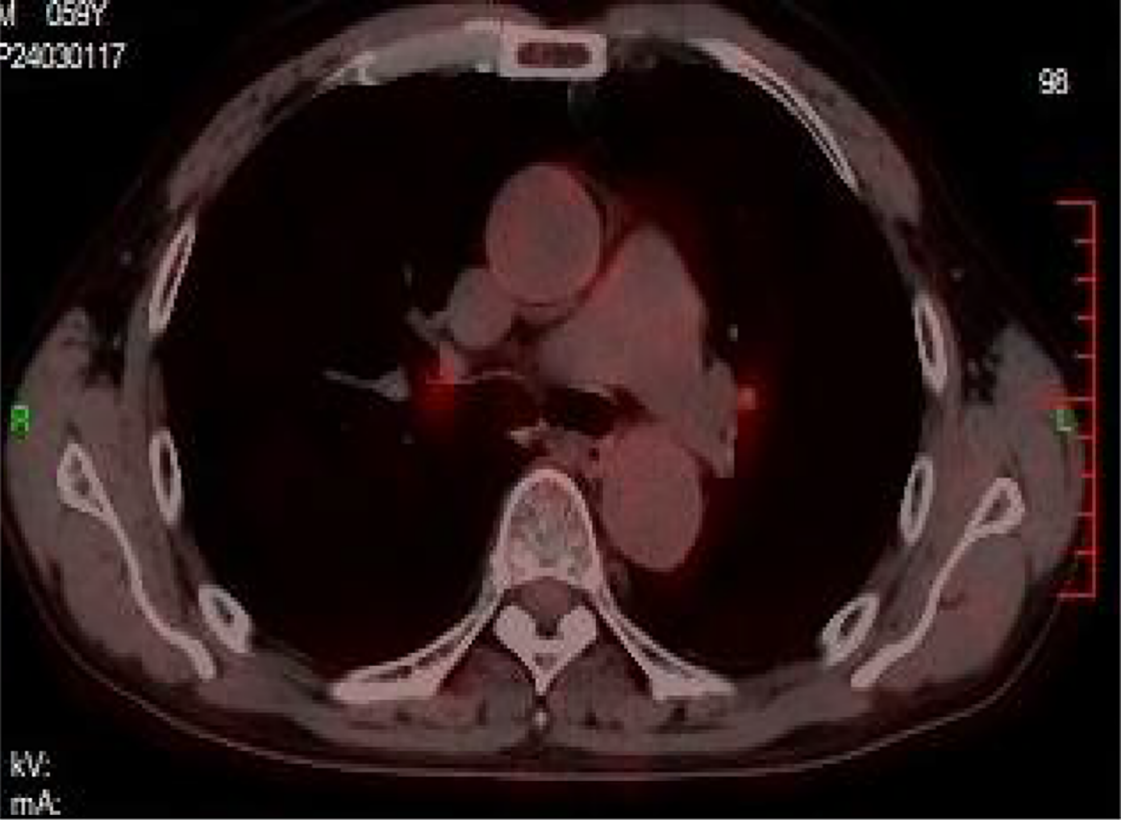
PET-CT: No abnormal hypermetabolic foci were seen in the pulmonary artery tract.

Auxiliary tests: blood gas analysis: pH: 7.41, pCO2: 28 mmHg, pO2: 112 mmHg, SO2 98%, D-dimer: 1,640.39 ng/ml, N-terminal cerebral natriuretic peptide precursor: 361.31 pg/ml; leukocytes: 16.02 × 10^9/L, neutrophil percentage 94%, ultrasensitive C-reactive protein 29.22 mg/L, calcitonin 1.31 ng/ml, creatinine: 208.0 umol/L; Electrocardiogram (ECG): sinus rhythm Abnormal EKG I, avl, V1\R\V6 leads T-wave is low and inverted. Cardiac ultrasound (Figure 1): EF 59%, the patient's arterial duct is not fully closed and there is a left-to-right blood shunt with a maximum velocity of the shunt of approximately 440 cm/s. There is a 21 × 14 mm bulge in the left pulmonary artery. Enlarged left atrium (39 mm anteroposterior diameter), hypodiastolic left ventricle. The right atrium and right ventricle had no abnormalities and mild mitral regurgitation. CTA of chest pain diathesis (Figure 2): nodular low-density filling defects are seen in the lumen of the left pulmonary arterial trunk in close relation to the ductus arteriosus. The left pulmonary artery trunk is altered, and PE is considered a high possibility. Lower extremity venous ultrasound: right calf intermuscular vein thrombosis (acute phase.) PET-CT (Figure 3) suggests: no abnormal hypermetabolic foci in the pulmonary artery tract. Blood culture (anaerobic and aerobic): human staphylococcus. Admission diagnosis: pulmonary artery occupational lesion to be investigated. After admission, the patient was treated with sodium ampicillin (2.0 g Q12H) for infection, heparin sodium (5,000 IU Q12H) for anticoagulation, and metoprolol succinate extended-release tablets (23.75 mg QD) to correct cardiac remodeling, among other treatments, and his condition was relieved. Later, to further clarify the diagnosis, on the 9th day after admission, the patient underwent cardiac surgery with pulmonary artery ligation and pulmonary artery resection and extracorporeal circulation-assisted open cardiac surgery, and the pulmonary artery organisms were sent to the pathology after the surgery, which suggested normal blood clots. After 16 days of hospitalization, the patient was discharged from the hospital after his condition improved. Upon discharge, the patient was prescribed rivaroxaban 10 mg daily and digoxin 0.25 mg daily. During telephone follow-ups one week and two weeks after discharge, the patient reported a significant relief of dyspnea. Twenty days later, during an outpatient follow-up, a cardiac ultrasound: EF 65%, after arterial catheter ligation, after resection of pulmonary valve redundancy, with mild tricuspid regurgitation. Regular follow-up of patients in outpatient clinics. Reviewing the patient's medical history again, the patient did not have chest pain, fever and other discomforts, cardiac ultrasound suggested arterial duct failure, chest pain dichotomy CTA showed that the nodular low-density filling defect in the trunk of the left pulmonary artery was closely related to the ductus arteriosus, and PET-CT did not show show significant hypermetabolism of the pulmonary arteries. The pulmonary artery occupying lesion was considered to be a PAIST formation due to arterial ductus arteriosus, endothelial injury due to sustained high flow and pressure at the main pulmonary artery junction, and increased vascular shear.

## 3 Discussion

PAIST refers to pulmonary arterial system *ab initio* thrombosis, meaning pulmonary artery (PA) occlusion occurs independently of peripheral thrombosis (1, 2, 11, 12). Hence, a case of isolated PE without deep vein thrombosis (DVT) is likely indicative of PAIST (1). However, the presence of peripheral thrombus does not rule out PAIST, as demonstrated in this case of PAIST with acute thrombosis of the deep veins in the right lower extremity.The specific etiology and pathogenesis of PAIST formation is unknown (2), and it has been reported that lung injury, infection, trauma, congenital heart disease, pulmonary hypertension(PH), radiotherapy, white stuffing syndrome, platelet activation, cytokine-mediated immunopathological response, and intravascular hemolysis can lead to the formation of PAIST (1, 2, 11–15), meanwhile, the thrombotic potential of Covid-19 has been recognized (8, 14, 15). PAIST originates in a hypoxic and inflammatory environment, leading to pulmonary vascular endothelial cell dysfunction in response to various factors such as disease, injury, and medications. This dysfunction results in an imbalance between thrombosis and fibrinolysis (1, 2). Endothelial dysfunction and platelet activation are key mechanisms involved in *in situ* thrombosis (13), along with age-dependent factors and neutrophil involvement (12, 16). In this case, the patient was a middle-aged male with a history of congenital heart disease in which arterial ductus arteriosus caused sustained high flow and pressure at the main pulmonary artery junction, and finally pulmonary artery endothelial cell damage, which impaired the antithrombotic function of the arterial wall by down-regulating the endothelial nitric oxide synthase (eNOS) or stimulating the expression of the adhesion receptor (8, 13), and, thus, formation of an *in situ* thrombus at the junction out of the main pulmonary artery.

The clinical manifestations of PAIST are nonspecific and similar to those of PE (Table 1), which may manifest as dyspnea, chest tightness, chest pain, palpitations, fever, hemoptysis, and other symptoms, and are less likely to cause right heart dysfunction, whereas PE often has right atrial and right ventricular changes, but it remains difficult to differentiate between the two in terms of symptoms (1–3). In this case, the patient presented with dyspnea, but without any right atrial or right ventricular changes or pulmonary hypertension (PH), which provides little support for the diagnosis of PAIST.

**Table 1:** Differential diagnosis of PAIST and PE.

	PAIST	PE
Symptomatic	Symptoms such as dyspnea, chest tightness, chest pain, palpitations, fever, hemoptysis, etc., less likely to cause right heart dysfunction	Dyspnea, chest tightness, chest pain, palpitations, fever, hemoptysis and other symptoms, often causing right heart dysfunction
Etiology	Lung injury, infection, trauma, congenital heart disease, etc.	peripheral thrombosis
Pathogenesis	Pulmonary artery endothelial cell damage, increased blood viscosity, increased blood flow velocity, inflammation and so on	Abnormal blood flow, vascular endothelial damage, or hypercoagulability of blood
Embolism characteristics	Mostly solitary lesions, often occurring at sites of pulmonary artery anomalies or lesions	Multiple more than single, located in the lower lobes of the lungs more than the upper lobes
Site of occurrence	Common in peripheral arteries, nonocclusive and eccentric, obtuse angle to vessel wall, no vasodilation	Often centrally located (i.e., proximal to segmental branches), mostly occlusive, with vasodilatation and at an acute angle to the vessel wall
Curing	Anticoagulation, thrombolysis and surgical, anti-inflammatory, anti-platelet aggregation	Anticoagulation, thrombolysis and surgery
Prognosis	Good prognosis, may progress to chronic thromboembolic pulmonary hypertension	Low-risk and low-intermediate-risk have a better prognosis, intermediate-high-risk and high-risk have a worse prognosis and can progress to chronic thromboembolic pulmonary hypertension

PAIST is a rare condition that lacks specific diagnostic criteria and imaging manifestations, often leading to misdiagnosis as PE (1). However, understanding its pathophysiological features and utilizing appropriate imaging techniques can aid in accurate diagnosis (1). In PE, the distribution of emboli is typically multiple rather than single, and they are commonly found in the lower lobes of the lungs rather than the upper lobes. In contrast, PAIST tends to manifest as a single lesion, frequently occurring at the site of an anomaly or lesion in the pulmonary artery (5, 10). The imaging manifestations of PAIST typically present in peripheral arteries as non-occlusive and eccentric lesions, often at an obtuse angle to the vessel wall and without vasodilation. In contrast, pulmonary embolism (PE) commonly exhibits centrally located emboli (proximal to segmental branches), frequently occlusive, with vasodilation, and at an acute angle to the vessel wall (4, 11, 17, 18). Solated pulmonary embolism: imaging often shows pulmonary artery obstruction. PAIST: may not have obvious features of acute obstruction, imaging may show that the thrombus *in situ* is more tightly bound to the wall of the pulmonary artery, and the morphology and density features may vary. Thrombosed pulmonary arteries are often located in lung tissue with underlying pathology, such as inflammation or tumour, and tend to be located in the distal branches, usually not involving the main trunk of the pulmonary artery. Alternatively, as in this case, the thrombus may form near the pulmonary artery connected to the ductus arteriosus (1, 10–13). In this case, the patient's imaging revealed a single filling defect in the left pulmonary artery trunk, located near the arterial conduit, with an unobstructed distal artery. Additionally, the lung parenchymal lesion was adjacent to the artery. Consequently, despite the presence of lower-extremity venous thrombosis, we attributed the low-density filling defect in the pulmonary artery to PAIST formation.

PAIST has received limited study, and its treatment parallels that of PE, including therapeutic options like anticoagulation, thrombolysis, and surgery. Hemoptysis is one of the most frequent complications associated with PAIST (1, 2, 11). It has also been reported that the treatment of *in situ* PAT may depend on the location of thrombosis in the pulmonary arterial tree and aspects of the specific clinical situation (1, 13). Inhibition of platelet activity through antiplatelet agents decreases the likelihood of recurrent PA thrombotic events following discontinuation of anticoagulation therapy (13). Inflammation is recognized as one of the causative mechanisms of *in situ* thrombosis, suggesting potential benefits for patients with *in situ* thrombosis from anti-inflammatory therapy (1). We should decide whether to go for anticoagulation only after considering the patient's symptoms, bleeding risk, and comorbidities (1, 11).

Our patient was previously in good health and had a low risk of bleeding, and was admitted to the hospital and treated with anti-infection and anticoagulation, and then underwent cardiac surgical operation of vein catheter ligation and resection of pulmonary artery lesion and extracorporeal circulation-assisted open heart surgery. Since the rate of thrombus regression in patients with PAIST is slower than that in patients with pulmonary embolism (10). The patient was then advised to continue taking oral anticoagulant rivaroxaban upon discharge from the hospital, with the decision for anticoagulation guided by the findings of outpatient follow-up visits. PAIST generally carries a favorable prognosis, but delayed diagnosis and treatment may lead to progression to chronic thromboembolic pulmonary hypertension (CTEPH) (13).

## 4 Conclusion

The clinical manifestations of PAIST lack specificity, and there is no definitive diagnostic gold standard. The incidence is often underestimated, and it is prone to misdiagnosis as PE (1, 2, 11–13). Hence, when encountering a solitary lesion within the pulmonary artery in clinical practice, differentiation from *in situ* thrombosis is necessary. In particular, the presence of underlying lesions of the pulmonary arteries, in this case thrombosis in the vicinity of the pulmonary artery connected to the ductus arteriosus, aided the diagnosis. Further research on PAIST is warranted to better understand its imaging characteristics, natural progression, and its significance in different disease contexts, ultimately informing treatment strategies and improving patient outcomes.

## Data Availability

The original contributions presented in the study are included in the article/Supplementary Material, further inquiries can be directed to the corresponding author.
